# Cardiac Systolic and Diastolic Function in Relation to Cardiovascular Risk Factors: Comparing Strain Imaging in Russian and Norwegian Populations: Heart-to-Heart—Norwegian-Russian Multilevel Educational Collaboration in Cardiovascular Disease Epidemiology

**DOI:** 10.1016/j.cjco.2025.11.010

**Published:** 2025-11-20

**Authors:** Assami Rösner, Mikhail Kornev, Hatice Akay Caglayan, Sofia Malyutina, Andrew Ryabikov, Alexander V. Kudryavtsev, Henrik Schirmer

**Affiliations:** a Department of Cardiology, Division of Cardiothoracic and Respiratory Medicine, University Hospital of North Norway, Tromsø, Norway; b Department of Clinical Medicine, UiT The Arctic University of Norway, Tromsø, Norway; c Research Institute of Internal and Preventive Medicine, Branch of the Institute of Cytology and Genetics, Russian Academy of Sciences Siberian Branch, Novosibirsk, Russia; d Novosibirsk Medical Institute, Novosibirsk, Russia; e International Research Competence Centre, Northern State Medical University, Arkhangelsk, Russia; f Department of Cardiology, Akershus University Hospital, Lørenskog, Norway; g Institute of Clinical Medicine, Cardiovascular Research Group, Campus Ahus, University of Oslo, Oslo, Norway

**Keywords:** Systolic function, Diastolic function, Strain imaging, Echocardiography, Comparison of the Russion and Norwegian population

## Abstract

**Background:**

Rates of cardiovascular morbidity and mortality are high in Russia. We compared conventional echocardiography and strain-based measures between Russian and Norwegian populations, and examined associations between hemodynamic and risk factors.

**Methods:**

Echocardiography was performed in 1192 participants from Arkhangelsk or Novosibirsk (Russia), and 917 from Tromsø (Norway), aged 40–69 years. “Normal” was defined as the absence of hypertension or cardiovascular disease. Conventional parameters and 2-dimensional speckle-tracking longitudinal strain and strain rate (systolic, early and late diastolic) were analyzed. Participants were categorized into 4 groups: normal, controlled hypertension, hypertensive blood pressure, and cardiac disease. Between-population comparisons used linear regression adjusted for prespecified covariates: age, sex, height, body mass index, blood pressure, heart rate, atrial fibrillation, smoking, pulmonary hypertension, and serum biomarkers (lipids, triglycerides, creatinine, high-sensitivity C-reactive protein, and HbA1c).

**Results:**

Russians showed a tendency toward lower longitudinal systolic functional indices, most evident in the normotensive group; however, these differences became nonsignificant after adjustment for hemodynamic and clinical covariates. Russians had lower stroke volume, higher heart rate, larger left atrial volume, and higher E/A ratio, indicating more impaired relaxation and elevated filling pressures. Groupwise comparisons (normal, controlled hypertension, hypertensive blood pressure, cardiac disease) showed that diastolic differences persisted after multivariable adjustment.

**Conclusions:**

After accounting for factors that influence cardiac function, systolic longitudinal strain parameters did not differ between Norwegian and Russian populations. In contrast, diastolic abnormalities persisted after adjustment, suggesting residual confounding and/or unmeasured determinants of diastolic dysfunction in the Russian population.

## Résumé

**Contexte:**

Les taux de morbidité et de mortalité cardiovasculaires sont élevés en Russie. Nous avons comparé les paramètres basés sur l’échocardiographie conventionnelle et la mesure de la déformation du muscle cardiaque (*strain*) entre les populations russe et norvégienne, et examiné les liens entre facteurs hémodynamiques et facteurs de risque.

**Méthodologie:**

Une échocardiographie a été réalisée chez 1 192 participants d’Arkhangelsk/de Novossibirsk (Russie) et chez 917 de Tromsø (Norvège) âgés de 40 à 69 ans. La « normalité » était définie comme l’absence d’hypertension ou de maladies cardiovasculaires. Les paramètres classiques ont été analysés, ainsi que la déformation longitudinale et la vitesse de déformation (systole et en début/fin de diastole) du muscle cardiaque, en deux dimensions, à l’aide de la méthode de suivi des marqueurs acoustiques (*speckle tracking*). Les participants ont été classés en quatre groupes : pression artérielle normale, hypertension maîtrisée, hypertension et maladie cardiaque. Les comparaisons entre les populations reposaient sur une régression linéaire ajustée pour tenir compte de covariables préétablies : âge, sexe, taille, indice de masse corporelle, pression artérielle, fréquence cardiaque, fibrillation auriculaire, tabagisme, hypertension pulmonaire et biomarqueurs sériques (lipides, triglycérides, créatinine, protéine C réactive hautement sensible et hémoglobine glyquée [HbA_1c_]).

**Résultats:**

Les indices fonctionnels systoliques longitudinaux ont eu tendance à être plus bas chez les Russes, une différence qui a été la plus marquée dans le groupe normotendu. Cette différence a néanmoins cessé d’être significative après ajustement pour tenir compte des covariables hémodynamiques et cliniques. Les Russes ont présenté un volume d’éjection plus faible, une fréquence cardiaque plus élevée, un volume de l’oreillette gauche plus important et un rapport E/A plus élevé, ce qui indique une altération plus grande de la relaxation du muscle cardiaque et des pressions de remplissage plus élevées. Les comparaisons par groupe (pression artérielle normale, hypertension maîtrisée, hypertension, maladie cardiaque) indiquent que les différences diastoliques ont persisté après l’ajustement multivarié.

**Conclusions:**

Après la prise en compte des facteurs qui influencent la fonction cardiaque, les paramètres de la déformation systolique longitudinale n’étaient pas différents entre les populations norvégienne et russe. En revanche, les anomalies diastoliques ont persisté après ajustement, ce qui donne à penser qu’il subsiste des facteurs de confusion et/ou des déterminants non mesurés de la dysfonction diastolique dans la population russe.

Cardiovascular morbidity and mortality rates are notably higher in Russia compared to developed Western European populations.^1^ Heart-to-Heart is a project designed to explore underlying causes for the high premature mortality and cardiovascular risk in Russia.^2^ The project draws comparisons between the Russian Know Your Heart (KYH) study and the seventh survey of the Norwegian Tromsø Study (Tromsø7), with the Norwegian population serving as the reference.

Previous Heart-to-Heart publications have found a higher prevalence of obesity, smoking, diabetes, and hypertension in the Russian population,^2^ conditions associated with reduced cardiac function.^3^

Factors such as blood pressure, diabetes, body mass index (BMI), age, and sex are known to affect both systolic and diastolic cardiac function.^4^^,^^5^

Strain rate (SR) imaging by 2-dimensional speckle-tracking echocardiography (STE) is a sensitive and specific method for detecting latent systolic and diastolic dysfunctions.^6^ In addition, global longitudinal strain identifies early signs of heart failure better than conventional echocardiographic parameters due to reduced dependence on geometric assumptions, and better reproducibility, making them particularly useful in population-based studies.^7^

This study aims to compare conventional echocardiography and strain and SR (S/SR) parameters between the Norwegian and Russian populations and how these echocardiographic parameters relate to risk factors potentially contributing to variation in cardiac function.

## Methods

### Study population

This study was based on 2 cross-sectional population-based studies conducted at 3 locations. First, the KYH study was conducted in Arkhangelsk and Novosibirsk between 2015 and 2018. The study recruited 5088 women and men aged 35-69 years for the baseline interview, and 2381 participants from Arkhangelsk and 2161 from Novosibirsk underwent health checks. Second, the Tromsø7 study, conducted in 2015 and 2016, invited all citizens in the Tromsø municipality aged ≥ 40 years, of whom 21,083 women and men aged 40–99 years (64.7%) participated.^8^ The KYH and Tromsø7 studies were planned in parallel by collaborative research groups with harmonized study questionnaires, procedural protocols, and protocols for echocardiographic examinations. Echocardiography was performed in prespecified subsamples of the KYH and Tromsø7 studies. Baseline characteristics of participants with and without echocardiographic examinations have been reported in the parent cohort publication,^2^ demonstrating that those examined were broadly representative of the full study populations. For the present substudy, S/SR parameters were analyzed in all echocardiograms with adequate image quality, and no further selection was applied.

**Figure 1:**
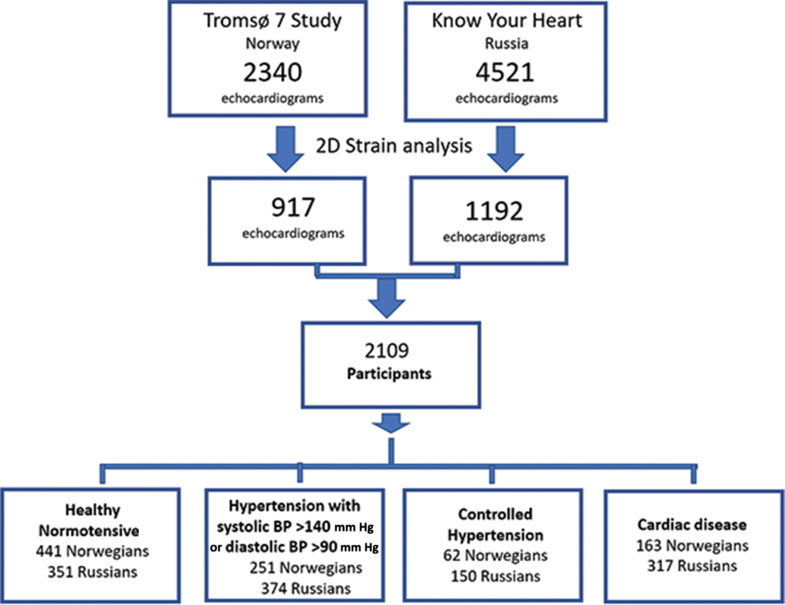
Flowchart detailing the inclusion and exclusion procedures. BP, blood pressure. Tromsø 7, Norwegian Tromsø Study; 2D, 2-dimensional.

Among the participants who underwent echocardiography, a random study sample of equal-sized age groups (40-49, 50-59, and 60-69 years) and sex groups was selected (N = 2109), comprising residents of Arkhangelsk (n = 595), Novosibirsk (n = 597), and Tromsø (n = 917), a randomly selected subgroup enabling the time-consuming measurements, semi-automated tracking, and strain analyses. After quality control, only echocardiograms with adequate image quality were analyzed. The flowchart in Figure 1 shows the selection of participants.

### Data collection

All selected participants from the KYH and Tromsø7 studies underwent medical examination, including echocardiography, questionnaires, and biological sample collection. Transthoracic echocardiography was performed in the left lateral decubitus position using a commercially available GE Healthcare Vivid q system (Horton, Norway) equipped with a 1.5-3.6 MHz sector matrix transducer in Russia, and a Vivid E9 machine with a single crystal matrix sector probe of 1.5-4.6 MHz in Norway. In both studies, conventional 2-dimensional greyscale images and pulsed, continuous, and colour Doppler data were acquired from parasternal and apical views. For the subsequent S/SR analysis, greyscale images were obtained at a frame rate of at least 50 frames per second. Additionally, an EchoPAC (v.203, GE-Vingmed AS, Horten, Norway) was used for offline conventional and S/SR measurements. Trained echocardiography specialists performed all examinations. Conventional measurements were assessed regularly within and between the reading laboratories in Novosibirsk and Tromsø. To avoid systematic errors, we replaced left atrial (LA) diameter, basal tissue Doppler velocities, and mitral annular plane systolic excursion (MAPSE) with LA volume, STE-derived basal velocities, and mitral annular displacement measurements from a single independent reader. The ejection fraction (EF) was calculated using Simpson’s method, and pulmonary hypertension was defined as an uncorrected tricuspid regurgitation gradient > 35 mm Hg.

### S/SR analysis

Ventricular strain measurement data were obtained in apical 2 chamber (2CH) and 4-chamber (4CH) views. All S/SR analyses (Q-analysis, EchoPAC, GE-Vingmed AS) and atrial volume measurements were performed by a single independent reader blinded to the original site echocardiographic measurements and reports. These measurements were therefore available for only interobserver comparison to other core-laboratory readers in Tromsø. By contrast, conventional echocardiographic parameters such as volumes, EF, and Doppler-based indices were assessed onsite and compared across laboratories to evaluate systematic differences.

The myocardial borders were manually traced, and the region of interest was corrected for myocardial thickness. Peak R was selected to define the time-point of end-diastole, whereas end-systole (ES) was defined as the timepoint of aortic valve closure (AVC). Left ventricular systole was measured from the peak of the R-wave to AVC, and diastole was defined as the time between AVC and peak R on spectral tracing of Doppler flow.

The automatically administered timepoints for AVC were manually corrected when necessary. After automated tracking, the software extracted the longitudinal ES segmental mid-myocardial strain, peak systolic SR (SR S), peak diastolic SR E (at early diastole), and SR A (during atrial contraction), and after discarding segments with strain-curve artefacts^3^ segmental S/SR averages were calculated for each heart. The STE-derived peak velocity and displacement values of the 4 basal segments were averaged to calculate the basal displacement and basal peak velocities during systole (s’), early diastole (e’), and atrial contraction (a’).

Artefact detection was performed by a second independent reader using screenshots of the strain curves, and images with apical foreshortening and curved artefacts were excluded from the analyses. Strain-curve artefacts were defined as curves deviated in diastole, blunted curves that showed reduced strain with missing post-systolic strain (PSS), or floating curves with deformations unrelated to the curves of other segments.^3^

### Definition of subgroups

The study sample was divided into the following 4 subgroups: participants with cardiac disease, participants with hypertension (blood pressure [BP] > 140/90 mm Hg), participants with controlled hypertension, and participants who were normotensive (Fig. 1).

Cardiac disease was defined as presence of the following criteria: elevated N-terminal pro-B-type natriuretic peptide (NT-proBNP) level according to age- and sex-specific limits,^9^ Q-wave (classes 1.1-1.2.7. of the Minnesota Code), left bundle branch block, history of myocardial infarction or heart attack, EF < 45%, aortic regurgitation, mitral regurgitation or mitral stenosis (grade 3 and 4), moderate aortic stenosis (mean pressure gradient > 25 mm Hg), and peak tricuspid regurgitation gradient > 30 mm Hg. Valvular stenosis was graded by valvular gradients and areas, and we used a multiparametric, semiquantitative approach for valvular regurgitation, as recommended in the guidelines.^10^

Following the 2024 European Society of Cardiology hypertension guidelines,^11^ hypertension was defined as systolic blood pressure ≥ 140 mm Hg or diastolic blood pressure ≥ 90 mm Hg, as measured during the health check. Controlled hypertension was defined as a BP below these thresholds, in combination with self-reported use of antihypertensive medications. Healthy normotensive participants were those for whom the criteria for cardiac disease or hypertension did not apply.

### Statistical analyses

We performed 1-way analysis of variance (ANOVA) with Bonferroni post hoc tests to assess between-group differences in continuous echocardiographic parameters. A χ^2^ test was used for group comparisons of categorical variables. Linear regression analysis was used for comparisons of continuous variables with and without adjustment for covariates.

All statistical analyses were performed using SPSS version 28.0 (IBM, Armonk, NY), and statistical significance was set at a 2-sided *P* < 0.05. Continuous data are presented as mean (M) ± standard deviation (SD), and NT-proBNP is presented as a skewed variable as the median (Me) with upper and lower quartiles (Q1; Q3). Categorical characteristics are presented as absolute numbers (Abs) and proportions (%).

Intraobserver variability in S/SR measurements was calculated from 135 randomly selected echocardiographic records comprising 1620 segments. These were reanalyzed using the same observer 6-12 months after the initial analysis. Subsequently, a second observer trained in the same echocardiography laboratory reanalyzed the same images for interobserver variability. Both observers performed at least 500 readings before performing the intraobserver and interobserver studies. For the interobserver variability of conventional systolic and diastolic parameters, 40 randomly selected echocardiograms from Russia and Norway were reanalyzed by the main readers of the Russian and Norwegian reading laboratories who were blinded to the compared results. Intraobserver and interobserver variability were assessed as the limits of agreement in Bland-Altman plots, and intraclass correlations are described in more detail in previous publications.^3^^,^^12^

## Results

Table 1 presents general demographic differences between the Russian and Norwegian populations. Supplemental Table S1 additionally illustrates differences between the 2 Russian populations from Arkhangelsk and Novosibirsk. Although the dataset was stratified by sex and age groups, Norwegian participants were slightly, but not significantly, older than Russian participants. Additionally, Norwegian participants were significantly taller, with a lower percentage showing an elevated BMI. BMI was highest in the Novosibirsk group. Russian participants had higher systolic and diastolic BP. Concerning other cardiovascular risk factors, Norwegians displayed lower low-density lipoprotein (LDL) cholesterol levels, and the percentage of lipid-lowering drug usage was significantly lower among them (Table 1). In addition, diabetes, daily smoking, renal failure, and elevated NT-proBNP levels were less prevalent in the Norwegian population.

**Table 1:** Characteristics of Norwegian and Russian participants

Characteristic	Russian	Norwegian	*P*
	Mean ± SD or *n* (%)	Mean ± SD or *n* (%)	
Group *n*	1192	917	
Women	594 (50)	460 (50)	0.987
Men	598 (50)	457 (50)	
Age, y	54.9 ± 8.5	56.0 ± 8.5	0.012
Height, cm	168 ± 9.5	172 ± 9	**< 0.001**
BMI, kg/m^2^	28.1 ± 5.7	27.2 ± 4.5	**< 0.001**
Systolic BP, mm Hg	133 ± 20	129 ± 20	**< 0.001**
Diastolic BP, mm Hg	84 ±11	76 ± 11	**< 0.001**
LDL cholesterol, mmol/L	3.73 ± 0.91	3.6 ± 0.9	**0.001**
Cholesterol, mmol/L	5.5 ± 1.1	5.5 ± 1.0	0.311
Triglycerides	1.58 ± 1.2	1.48 ± 0.9	**0.039**
Lipid-lowering drugs	256 (21.5)	147 (16.3)	**< 0.001**
HbA1c, %	5.6 ± 0.5	5.6 ± 0.5	0.977
Diabetes	82 (6.9)	37 (4.0)	**0.019**
Smoking daily∗	302 (25.4)	121 (13.2)	**< 0.001**
Creatinine, μmol/L	87 ± 29	74 ± 15	**< 0.001**
HS-CRP, mg/L	3.6 ± 7.3	1.7 ± 2.0	**< 0.001**
Atrial fibrillation	45 (3.8)	32 (3.5)	**0.005**
Hx of renal failure	241 (20.2)	34 (3.8)	**< 0.001**
Hx of cancer	81 (6.7)	66 (7.4)	0.873
Hx of asthma	60 (5.0)	109 (12.2)	**< 0.001**
Hx of stroke	38 (3.2)	21 (2.3)	**0.049**
NT-proBNP, pmol/L	172 (14.4)	46 (28/59)	**0.005**
Valvular heart disease ≥ grade II	7 (0.6)	2 (0.2)	0.396
High LA volume index	160 (13.4)	91 (9.9)	**< 0.001**
BP medication	446 (37.4)	162 (17.7)	**< 0.001**

Boldface indicates significance. High N-terminal pro-B-type natriuretic peptide (NT-pro BNP) level: age and gender and population-based cutoff values; high body mass index (BMI), ≥ 30 kg/m^2^; high low-density lipoprotein (LDL) cholesterol, ≥ 3.0 mmol/L; high hemoglobin (Hb)A1c, ≥ 6.5%; high LA volume index, > 34/mL/m^2^.

BMI, body mass index; BP, blood pressure; HDL, high-density lipoprotein; HS-CRP, high-sensitivity C-reactive protein; Hx, history; LVEF, left ventricle ejection fraction; SD, standard deviation.

∗ Refers to active current smoking; NT-proBNP level in median (lower quartiles/upper quartiles).

The Norwegian population also had fewer participants with a history of angina, heart attack, or heart failure. The marginally higher prevalence of atrial fibrillation in the Russian population (3.8% vs 3.5%; Table 1) might also have contributed to the significantly larger atrial volume index (LAVI) observed, but the small difference in prevalence makes it unlikely to represent more than a minor contributing factor. The differences in antihypertensive drug use also are shown in Table 1.

To assess cardiac functional parameters, we compared the Russian and Norwegian subpopulations within their respective subgroups. The differences between the subgroups are presented in Table 2. Supplemental Table S2 shows the mean values of these measurements. Notably, in the normal and hypertensive subgroups, the mean heart rate was higher in the Russian participants than in the Norwegian participants, although most of the adjusted systolic parameters, such as stroke volume, longitudinal displacement, velocity, and strain, were not significantly different between the Russian and Norwegian participants.

**Table 2:** Mean difference between Norwegians and Russians for systolic and diastolic functional parameters

Parameter		Healthy normotensive Group A	Hypertension Group B	Controlled hypertension Group C	Cardiac disease Group D
	Group *n* (Norwegians/Russians)	441/351	251/374	62/150	163/317
		Mean difference Norwegians - Russians (95% CI)			
Longitudinal ES strain, %	Unadjusted	–0.15 (–0.49 to 0.19)	**–0.89 (–1.37 to –0.41)**	**–0.90 (–1.66 to –0.13)**	0.06 (–0.66 to 0.79)
	Adjusted	0.16 (–0.35 to 0.68)	–0.25 (–0.50 to 1.00)	–0.38 (–1.44 to 0.67)	–0.23 (–0.76 to 1.23)
Longitudinal peak SR S	Unadjusted	0.00 (–0.03 to 0.03)	–0.03 (–0.07 to 0.00)	–0.05 (–0.11 to 0.00)	0.01 (–0.04 to 0.05)
	Adjusted	–0.01 (–0.05 to 0.03)	–0.02 (–0.07 to 0.04)	–0.02 (–0.07 to 0.10)	–0.03 (–0.10 to 0.04)
Longitudinal peak SR E	Unadjusted	–0.02 (–0.07 to 0.03)	**0.06 (–0.01 to 0.11)**	–0.01 (–0.10 to 0.09)	–0.01 (–0.08 to 0.07)
	Adjusted	–0.00 (–0.06 to 0.08)	–0.05 (–0.13 to 0.04)	–0.06 (–0.20 to 0.08)	0.04 (–0.06 to 0.13)
Longitudinal peak SR A	Unadjusted	**–0.02 (–0.02 to 0.06)**	**–0.02 (–0.03 to 0.07)**	**0.03 (–0.05 to 0.10)**	**–0.02 (–0.08 to –0.04)**
	Adjusted	0.04 (0.03 to 0.09)	**0.08 (0.02 to 0.14)**	0.07 (0.03 to 0.16)	–0.17 (–0.09 to 0.06)
Longitudinal displacement, mm	Unadjusted	**0.81 (0.47 to 1.14)**	**1.14 (0.64 to 1.63)**	**1.41 (0.61 to 2.21)**	0.37 (–0.27 to 1.00)
	Adjusted	0.06 (–0.45 to 0.56)	0.06 (–0.71 to 0.84)	–0.29 (–0.9 to 0.1)	–0.09 (–0.97 to 0.79)
Peak velocity ś, cm/s	Unadjusted	–0.03 (–0.22 to 0.15)	0.07 (–0.21 to 0.36)	–0.35 (–0.14 to 0.84)	–0.04 (–0.29 to 0.37)
	Adjusted	–0.16 (–0.43 to 0.12)	–0.00 (–0.44 to 0.44)	0.09 (0.54 to 0.72)	–0.01 (–0.42 to –0.40)
Peak velocity é, cm/s	Unadjusted	**0.28 (–0.02 to 0.54)**	–0.15 (–0.51 to 0.22)	0.24 (–0.29 to 0.77)	–0.08 (–0.53 to 0.37)
	Adjusted	**0.49 (0.16 to 0.81)**	0.28 (–0.25 to 0.81)	0.01 (–0.71 to 0.73)	–0.41 (–0.95 to 0.13)
Peak velocity á, cm/s	Unadjusted	**–0.31 (–0.50 to –0.12)**	–0.08 (–0.32 to 0.16)	**–0.45 (–0.85 to –0.04)**	0.01 (–0.29 to 0.31)
	Adjusted	**–0.25 (–0.51 to–0.002)**	–0.23 (–0.57 to 0.11)	–0.53 (–1.1 to –0.02)	–0.05 (–0.37 to 0.48)
LA volume index, mL/m^2^	Unadjusted	**2.01 (1.03 to 2.99)**	–1.31 (–2.99 to –1.03)	–1.31 (–2.65 to 0.03)	–0.57 (–2.45 to 1.32)
	Adjusted	–0.05 (–0.12 to 0.01)	–0.08 (–0.17 to –0.01)	–0.10 (–0.25 to 0.05)	**–0.12 (–0.22 to –0.02)**
Heart rate, 1/min	Unadjusted	**–4.30 (–5.56 to –3.04)**	**–2.54 (–4.28 to –0.80)**	**–3.23 (–5.98 to –0.49)**	**–3.53 (–5.64 to –1.41)**
	Adjusted	**–4.77 (–6.69 to –2.85)**	–0.32 (–2.97 to 2.32)	**–4.18 (–8.10 to– 0.28)**	–2.03 (–4.78 to 0.73)
Stroke volume, mL	Unadjusted	**7.36 (4.44 to 10.3)**	**6.48 (2.76 to 10.21)**	**11.16 (5.46 to 16.86)**	**7.44 (3.04 to 11.84)**
	Adjusted	3.04 (–1.21 to 7.29)	4.21 (–1.42 to 9.83)	3.23 (–4.97 to 11.42)	2.39 (–4.13 to 8.90)
MV E/é	Unadjusted	0.05 (–0.48 to 0.57)	0.52 (–1.61 to 2.65)	0.93 (–0.40 to 2.25)	0.22 (–2.16 to 2.59)
	Adjusted	–0.64 (–1.49 to 0.20)	–0.07 (–3.53 to 3.39)	–1.38 (–0.75 to 3.50)	–0.95 (–2.55 to 4.45)
E/A ratio	Unadjusted	**–0.08 (–0.13 to –0.02)**	**–0.07 (–0.12 to –0.02)**	–0.10 (–0.20 to 0.00)	0.00 (–0.08 to 0.08)
	Adjusted	**–0.10 (–0.15 to –0.02)**	**–0.09 (–0.16 to –0.02)**	**–0.16 (–0.30 to –0.03)**	0.02 (–0.12 to 0.09)
MV E deceleration time, ms	Unadjusted	**–30.8 (–36.4 to–25.1)**	**–32.5 (–40.4 to –24.5)**	**–32.8 (–46.4 to–19.1)**	**–38.3 (–46.5 to –20.2)**
	Adjusted	**–32.4 (–41.2 to –23.6)**	**–32.0 (–44.2 to –19.7)**	**–43.2 (–63.7 to –22.7)**	**–38.8 (–49.6 to –26.1)**
Ejection fraction, %	Unadjusted	–0.81 (–0.76 to 0.60)	**–1.71 (–2.85 to 0.57)**	**–1.68 (–3.21 to –0.15)**	**–4.27 (–5.92 to –2.62)**
	Adjusted	–0.61 (–1.68 to 0.46)	**–3.18 (–4.93 to –1.42)**	–1.87 (–4.03 to 0.29)	**–3.68 (–5.99 to –1.38)**

Boldface indicates significance. Difference between Norwegians and Russians (N–R) unadjusted and adjusted for age, sex, body mass index, height, systolic blood pressure, diastolic blood pressure, heart rate, atrial fibrillation, smoking, pulmonary artery pressure, serum values for total, low-density lipoprotein and high-density lipoprotein cholesterol, triglycerides, creatinine, high-sensitivity C-reactive protein, and hemoglobin (HB)A1c. Heart rate was not adjusted for heart rate.

A: at atrial contraction; CI, confidence interval; E, early diastolic; ES, end-systolic; LA, left atrial; MV, mitral valve S, systolic; SR, strain rate.

**Figure 2:**
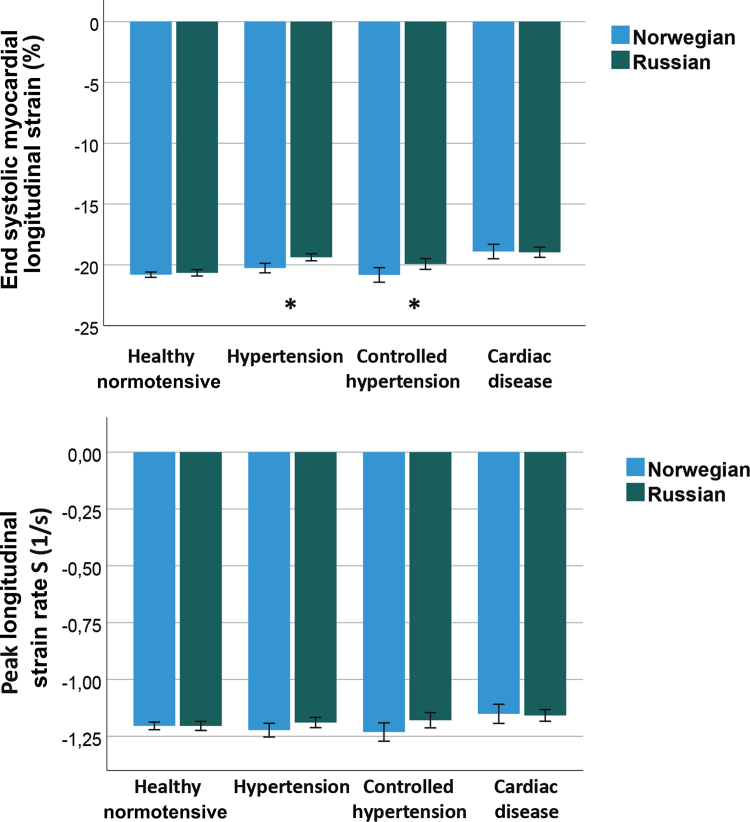
End-systolic (ES) global longitudinal strain comparing Russians and Norwegians in groups defined by blood pressure or heart disease. Elevated blood pressure (BP) was defined as systolic BP > 140 mm Hg or diastolic BP > 90 mm Hg. ∗*P* < 0.05 for unadjusted difference between Russians and Norwegians.

**Figure 3:**
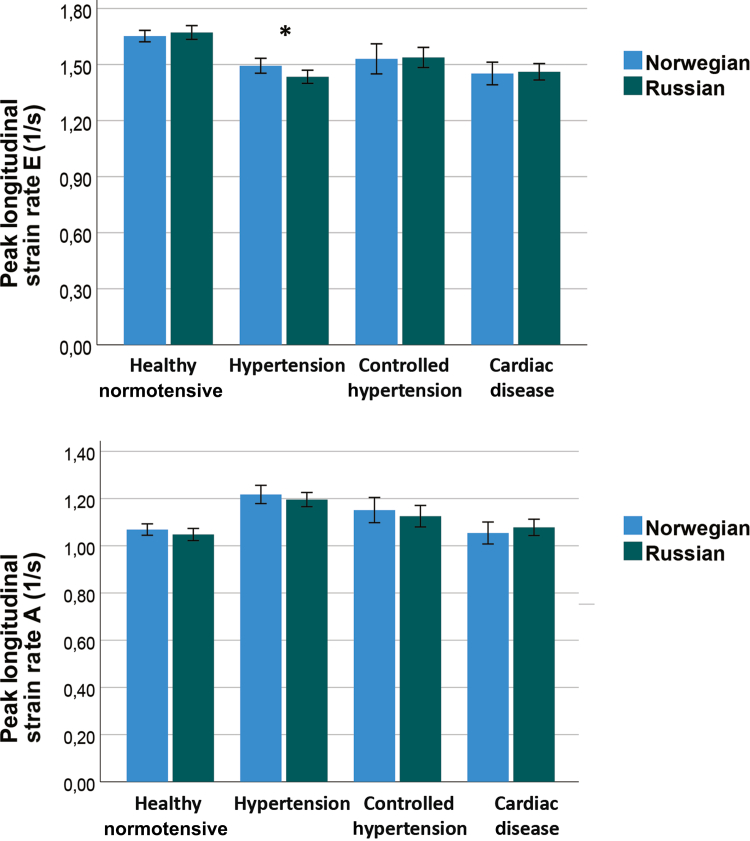
Global longitudinal early (E) diastolic strain rate (SR) and during atrial contraction (A) comparing Russians and Norwegians in blood pressure and heart disease groups. ∗*P* < 0.05 for unadjusted difference between Russians and Norwegians.

As shown in Figure 2, the unadjusted longitudinal systolic strain was slightly lower in the Russian population than in the Norwegian population, and only the 2 hypertension groups showed significant differences. Figure 3 shows that SR S, SR E, and SR A were largely similar across subgroups in the Russian and Norwegian populations, whereas unadjusted SR E was slightly but still significantly lower in the group with elevated BP than in the other groups. Left ventricular (LV) diastolic functional parameters based on conventional Doppler indices (Table 2) showed longer MV E DT and lower e’ potential indicators of impaired relaxation in the normal and hypertensive groups of the Russian population, with a longer MV E DT and higher LA volume index. Indicators of elevated filling pressure like higher E/e’ and higher E velocities were not significantly different between the adjusted groups. However, all parameters based on MV A and longitudinal function during the atrial contraction phase (ie, SR A, a’) were significantly lower in some groups of the Russian population.

Supplemental Table S3 presents the unadjusted and adjusted linear regressions for speckle tracking and conventional systolic and diastolic parameters. After adjustment, the systolic longitudinal functional parameters and SR E did not differ between the populations, and Russians still displayed reduced atrial SR A and velocities, as well as a higher LA volume. Regarding conventional parameters, E/A ratio, heart rate (HR), and EF were higher, E DT was longer, and SV was lower.

Supplemental Table S4 shows good interobserver agreement for global S/SR measurements, without significant differences between the readings of the different populations. However, Supplemental Table S5 demonstrates that the interobserver variability of the conventional echocardiographic parameters introduced a significant error, with “overestimation” of EF, MV deceleration time, and SV in the Russian population, whereas the readings of MV E/A ratios showed reliable intraclass correlation with insignificant deviation.

Supplemental Table S6 shows that systolic parameters were mainly influenced by afterload-related factors, such as body height, BMI, BP, HR, AF, and pulmonary hypertension, and general cardiovascular risk factors, such as LDL, high-density lipoprotein (HDL), creatinine, high-sensitivity C-reactive protein (HS-CRP) levels, and hemoglobin (HB)A1c were significant factors influencing diastolic function.

## Discussion

### Main findings of the study

In this study, we found significantly different cardiac functional properties between the Russian and Norwegian populations. After adjustment for covariates, the Russian participants still showed a lower SV and a higher HR. Diastolic properties with higher MV E DT and larger left atria indicated impaired relaxation, and higher MV E/A, and E/é indicate higher filling pressures. In contrast to longitudinal systolic parameters, diastolic early relaxation—reflected by a higher MV E DT or peak SR E—remained reduced in the Russian population after adjusting for known influencing factors. Russians also tended to have lower systolic functional parameters; however, longitudinal strain and tissue velocities did not differ after adjustment.

### Differences in cardiovascular risk factors and diseases

The Russian population has higher cardiovascular morbidity and mortality rates than developed Western European populations.^13^ Our findings confirm Russia’s unfavourable risk profile: participants had higher BP, higher levels of HS-CRP, higher BMI, higher diabetes rates, higher smoking rates, and higher rates of renal failure,^14^ identifying preventable causal factors for Russia’s high cardiovascular morbidity and mortality rates.^15^ Serum cholesterol levels were similar between the groups, but triglyceride levels were higher in Russians, likely reflecting the higher prevalence of diabetes. Cardiovascular disease was also more frequent, with higher rates of myocardial infarction and Q waves, elevated NT-proBNP level, and higher rates of stroke and pulmonary hypertension.^13^

A study on the Novosibirsk population confirmed that the prevalence of AF based on resting electrocardiograms was generally comparable to that of North American or European populations.^13^ Specifically, the proportions of paroxysmal AF and persistent AF were reported to be approximately 40% and 20%, respectively, in a Russian population cohort, and these conditions might be underdiagnosed. Additionally, individuals with AF are prone to both fatal and nonfatal cardiovascular events and could be underrepresented in the survey sample, due to selection bias. Such underrepresentation might explain the paradoxically low prevalence of AF reported in both populations.

### Systolic functional parameters, including global longitudinal strain and global longitudinal strain rate

Systolic S/SR parameters fall with high afterload and rise with increased contractility.^16^ Higher HR reduces ventricular filling and preload, whereas intracellular calcium accumulation can have the opposite effect. Elevated BP also reduces segmental and global S/SR.^13^ As BP medication use and cardiac disease were more common in the Russian population, we assessed whether functional differences were consistent across hypertension stages and disease status.

The effect of sex and age on S/SR are well known.^3^ In the present study, both populations were matched for age and sex, so these were not major explanatory factors.

A point that remains unclear is whether sex differences in S/SR are solely due to body size or also sex-specific differences. BMI directly influences vascular resistance and BP and may alter contractility through inflammatory or other unknown processes, further adding to S/SR differences.^16^ Additionally, age reduces longitudinal function,^3^ though not markedly in the age range of 40-69 years.^3^ In a previous study, normotensive individuals taking antihypertensive drugs had lower global strain than normotensive controls.^12^ In that study, hypertensive individuals had a higher HR and SV than normotensive individuals, whereas longitudinal S/SR and displacement were reduced, indicating that a higher SV relates to increased radial contraction.

In the present study, the Russian population had a higher HR and lower SV, indicating a higher sympathetic tone and cardio-depressive factors. As shown in Figures 2 and 3, Russian participants had slightly reduced strain and systolic SR, with only marginal uncorrected differences. The combination of higher S/SR with higher sympathic tonus and longitudinal S/SR reducing factors—higher BP, higher BMI, and shorter filling at high HR—narrowed interpopulation differences. Thus, differences in age, sex, height, BMI, HR, and BP likely explain the lower level of cardiac function in the Russian population. Moreover, most systolic longitudinal functional S/SR ratios were not significantly different after adjustment. Other factors, such as cholesterol, triglycerides, renal function, and HS-CRP level, had no direct influence on LV longitudinal function. Therefore, as the uncorrected strains showed the highest differences in the hypertensive groups, this pattern is consistent with an influence of elevated BP on longitudinal systolic and diastolic function; however, given the cross-sectional design, causality cannot be inferred.

### Diastolic functional parameters

Higher E/A ratios indicate higher filling pressures linked to higher HR and lower SV. Higher é and SR E may result from elevated filling pressures, whereas lower é or SR E can reflect impaired relaxation. Therefore, the combination of reduced relaxation and increased filling pressures likely explains the small effect of population differences on SR E. Beyond age, sex, BMI, BP, HR, and AF, which strongly affected systolic parameters, cholesterol, triglycerides, creatinine, HS-CRP, and HbA1c also influenced diastolic function, EF, SV, and HR. Diastolic function appears similarly affected by markers of diabetes, renal function, or inflammation, as well as by changes in pre-load and after-load.

Alcohol consumption is associated with impaired relaxation and elevated filling pressures.^14^ Differences in alcohol intake between Russian and Norwegian populations, although unmeasured, may contribute to diastolic variation. A recent KYH substudy of patients diagnosed with chronic alcohol use showed an association of é, E/e’, and LA volume with alcohol intake.^14^ Another KYH subanalysis linked binge drinking and sessional alcohol amount to diastolic function (positive for E/e’ and negative for e’) in a general population.^17^ In addition, psychosocial factors influencing sympathetic tone may play a role in these differences. The Novosibirsk population includes less than 5% individuals of non-Russian or Ukrainian ethnicity, a proportion too small to consider ethnicity a major confounder.

## Methodological considerations on interreader variability

To avoid inter-laboratory bias, the S/SR analyses and atrial volumes were read by a single independent reader. Similarly, the tissue Doppler imaging velocities were substituted with the STE peak values from basal segmental velocities. The conventional Doppler parameters were measured in separate laboratories in Russia and Norway. However, as shown in Supplemental Table S5, interobserver variability for MV Doppler-based parameters was excellent; therefore, this was unlikely to introduce bias. Furthermore, MV DT, even with minor systematic error, remained significantly longer in the Russian population. This finding is consistent with larger LA volumes in the Russian population, indicating delayed relaxation. Additionally, Doppler readings with large MV A differences between populations correlated well with A velocities and SR A, indicating higher filling pressures.

Therefore, differences in conventional diastolic parameters between populations were not attributable to inter-reader variability. Applying potential systematic reading errors to Supplemental Tables S2 and S3 suggests that EF was significantly lower in the Russian population. Another possibility is that the lower SV was underestimated. Lower EF correlated well with lower SV; however, these 2 parameters displayed the highest interreader variability, and they should therefore be interpreted with caution.

### Limitations

Selection bias might have played a considerable role in this study, as Russian and Central European individuals could plausibly exhibit different behaviours regarding participation in epidemiologic studies. Thus, selection bias due to self-recruitment cannot be excluded; the extent and direction of this bias may differ between populations. In addition, regional and recruitment differences between Arkhangelsk and Novosibirsk are known.^2^

Ventricular dimensions derived from M-mode echocardiograms, such as septal thickness, myocardial mass, end-diastolic diameter, and tissue Doppler measurements, showed high systematic differences between the Russian and Norwegian reading groups and were therefore not included in the present study. However, we included EF, SV, and MV DT, parameters known for high interreader variability, and the analyses should therefore be interpreted with caution. EF and SV were the only systolic parameters with significant differences between the groups, which may indicate a systematic reading error rather than true cardiac functional differences. Previously, Iakunchykova et al. investigated differences in biochemical markers and suggested adjustments for different laboratories; however, we used the original values,^18^ as biomarkers were used only for adjustment and description, making it unlikely that the correction would have changed the outcome.

Hypertension thresholds followed the 2024 European Society of Cardiology guidelines. Blood pressure strongly influences cardiac systolic and diastolic function and might also affect participants with high-normal blood pressure, defined as systolic BP of 130-139 mm Hg and diastolic BP of 85-89 mm Hg, who were categorized as normal in the present study. However, we adjusted for BP as a continuous variable across all 4 functional groups. Although multivariable adjustment was performed for major cardiovascular risk factors (including smoking, hypertension, obesity, and diabetes), residual confounding remains possible.

We assume that alcohol consumption and drinking patterns may have a considerable influence, particularly on diastolic cardiac function.^14^ The World Health Organization reports a per-capita alcohol consumption of 5.8 and 18.7 litres in women and men, respectively, in Russia, compared to 3.2 and 11.6 litres for women and men, respectively, in Norway.^19^^,20^ In the Russian population, alcohol consumption may be an important factor causing higher mortality and heart failure rates. However, harmonized alcohol consumption data were not available across cohorts; the only shared questionnaire item (“any alcohol use in the past year”) was too crude for meaningful adjustment. Thus, residual confounding by alcohol consumption patterns and amount is likely.^21^ Undetected ischemia may have contributed to observed diastolic abnormalities.

The extent and significance of differences between diastolic measurements across groups were not uniform. However, when comparing the Norwegian and Russian populations within each of the normal and hypertensive groups, we observed a consistent pattern of differences, suggesting that despite variability, meaningful comparisons are possible.

Selection bias also may have played a role due to recruitment being from only 2 urban regions in Russia (Arkhangelsk and Novosibirsk), which may limit the generalizability of our findings to the entire Russian population. Given the cross-sectional observational design, causal inferences cannot be made.

Comparable longitudinal studies for both populations were not available, which would have strengthened the study design. Future studies with harmonized alcohol assessments, stress imaging, and repeated echocardiography after structured atherosclerotic cardiovascular disease risk-factor control are warranted. Our findings should be considered hypothesis-generating rather than conclusive.

## Conclusion

We found that the Russian population showed lower levels of systolic and diastolic cardiac function compared to the Norwegian population. Higher BP, HR, BMI, sex, and age were the most important factors explaining the lower level of longitudinal function in the Russian population. However, even after further adjustment for known risk factors for suppressed longitudinal strain, including diabetes, cholesterol, and HS-CRP, a significant difference between the populations remained, indicating the possibility of a true underlying difference or contribution from residual confounders. Future studies are required to identify these additional risk factors.
