# Supplementary material for: Cardiac Systolic and Diastolic Function in Relation to Cardiovascular Risk Factors: Comparing Strain Imaging in Russian and Norwegian Populations: Heart-to-Heart—Norwegian-Russian Multilevel Educational Collaboration in Cardiovascular Disease Epidemiology
Source: CJC Open. 2025 Nov 20;8(3):296–306. doi: 10.1016/j.cjco.2025.11.010 (PMC12983276; doi:10.1016/j.cjco.2025.11.010)
Supplement: Supplemental Tables 1-6 [file mmc1.pdf]

Supplemental Table S1: Group characteristics of the three populations

	Arkhangelsk	Novosibirsk	Tromsø	
	Mean ± SD or *n* (%)	Mean ± SD or *n* (%)	Mean ± SD or *n* (%)	*p*-value
Group *n*	595	597	917	
Women	297 (50)	297 (50)	460 (50)	0.987
Men	298 (50)	300 (50)	457 (50)	
Age (years)	54.9 ±8.6	55.0 ±8.4	56.0 ±8.5	0.052
Height (cm)	168 ±9	168 ±9	172 ±9*†	**<0.001**
Weight (kg)	79 ±16	81 ±18	80 ±16	0.078
BMI (kg/m2)	27.8 ±5.3	28.5 ±6.0	27.2 ±4.5†	**<0.001**
High BMI (n)	179 (30.1)	206 (34.4)	214 (23.3) *†	**<0.001**
Systolic BP (mmHg)	133 ±20	134 ±20	129 ±20*†	**<0.001**
Diastolic BP (mmHg)	84 ±11	83 ±11	76 ±11*†	**<0.001**
LDL Cholesterol (mmol/l)	3.72 ±0.9	3.74 ±0.9	3.6 ±0.9*†	**0.006**
High LDL Cholesterol (n)	185 (31.1)	189 (31.6)	269 (29.3)	0.616
Cholesterol (mmol/l)	5.5 ±1.2	5.5 ±1.1	5.5 ±1.0	0.690
Triglycirides	1.53 ±1.2	1.63 ±1.2	1.48 ±0.9†	**0.046**
High Triglycerides (n)	77 (12.9)	109 (18.2)*	132 (14.4)	**0.035**
Lipid Lowering drugs (n)	143 (24)	113(19)	147 (16.3)*†	**<0.001**
HbA1C (%)	5.6 ±0.8	5.7 ±0.9	5.6 ±0.5	0.575
High HbA1C (n)	30 (5.0)	37 (6.2)	40 (4.4)	0.282
Diabetes (n)	42 (7.1)	40 (6.7)	37 (4.0)*	**0.019**
Smoking daily § (n)	128 (21.5)	174 (29.0)*	121 (13.2) *†	**<0.001**
Creatinin (mmol/l)	85 ±15	89 ±38*	74 ±15*†	**<0.001**
Creatinin high (n)	44 (7.4)	42 (7.0)	18 (2.0) *†	**<0.001**
HS CRP (mg/l)	3.1 ±4.9	4.0 ±9.0*	1.7 ±2.0 *†	**<0.001**
Hx of renal failure (n)	105 (17.6)	136 (22.9)*	34 (3.8) *†	**<0.001**
Hx of Cancer (n)	41 (6.9)	40 (6.7)	66 (7.4)	0.873
Hx of Asthma (n)	33 (5.5)	27 (4.5)	109 (12.2) *†	**<0.001**
Hx of Stroke (n)	25 (4.2)	13 (2.2)	21 (2.3)	**0.049**
NT-proBNP (pmol/l)	84 (48/152)	88 (41/165)*	46 (28/59)†	**0.005**
High NT-proBNP (n)	96 (16.1)	106 (17.7)	25 (2.7) *†	**0.005**
Valvular heart disease ≥ grade II (n)	3 (0.5)	4 (0.7)	2 (0.2)	0.396
LA volume index high (n)	112 (18.8)	48 (8.1) *	91 (9.9)*	**<0.001**

BMI: body mass index; BP: blood pressure; LDL: low density lipoproteins; HDL: high density lipoproteins; LV EF: left ventricle ejection fraction. §Refers to active current smoking; NT-proBNP in median (lower quartiles/upper quartiles)
Bonferoni post-hoc analysis
* p<0.05 for difference towards group A
† p<0.05 for difference towards group B

Supplemental Table S2: LV systolic and diastolic functional parameters

		Healthy Normotensives	Hypertension	Controlled hypertension	Cardiac Disease	
		Group A	Group B	Group C	Group D	
		Mean ± SD	Mean ± SD	Mean ± SD	Mean ± SD	ANOVA *p*-value
Group *n*	*Norwegians/ Russians*	441/351	251/374	62/150	163/317	
Longitudinal displacement (mm)	Norwegians	16.8 ±2.3	16.6 ±2.9	16.9 ±2.5	15.2 ±2.9 *†‡	<0.001
	Russians	16.0 ±2.2	15.5 ±2.8	15.5 ±2.6	14.7 ±3.2 *†	<0.001
Peak velocity s´ (cm/s)	Norwegians	5.5 ±1.3	5.2 ±1.6	5.4 ±1.5	5.2 ±1.4	0.092
	Russians	5.5 ±1.3	5.2 ±1.6 *	5.1 ±1.6 *	5.2 ±1.7 *	0.006
Peak velocity e´ (cm/s)	Norwegians	-7.0 ±1.8	-5.5 ±2.0 *	-5.7 ±1,6 *	-6.0 ±2.1 *	<0.001
	Russians	-7.2 ±1.7	-5.4 ±2.2 *	-6.0 ±1.7 *†	-6.0 ±2.2 *†	<0.001
Peak velocity a´ (cm/s)	Norwegians	-6.2 ±1.3	-6.7 ±1.3 *	-6.3 ±1.4	-6.0 ±1.4 †‡	<0.001
	Russians	-5.9 ±1.3	-6.6 ±1.4 *	-6.2 ±1.3†	-6.0 ±1.5†	<0.001
LA volume Index (ml/m^2)	Norwegians	24.5 ±6.7	24.6 ±7.9	24.6 ±7.9	25.1 ±10.5	0.908
	Russians	22.5 ±7.3	25.9 ±8.6 *	25.9 ±8.6 *	25.6 ±9.7 *	<0.001
Heart-rate	Norwegians	60 ±10	64 ±11 *	59 ±10†	62 ±11 *	<0.001
	Russians	64 ±9	67 ±11*	62 ±9*†	65 ±11‡	<0.001
Stroke volume (ml)	Norwegians	85.6 ±24.6	91.7 ±30.1 *	94.2 ±27.1	92.7 ±30.5 *	0.003
	Russians	78.3 ±14.7	85.2 ±17.1*	83.1 ±14.6*	85.2 ±18.3*	<0.001
MV E/e´(1/1)	Norwegians	10 ±3	12 ±5 *	12 ±5 *	12 ±6 *	<0.001
	Russians	10 ±3	14 ±8*	13 ±4 *	13 ±9 *	<0.001
MV velocity E (cm/s)	Norwegians	67 ±15	63 ±16 *	63 ±17	67±21	0.006
	Russians	69 ±17	64 ±16*	69 ±16†	66±19	0.002
MV A velocity (cm/s)	Norwegians	61 ±14	72 ±15 *	67 ±18 *	65 ±17 *†	<0.001
	Russians	58 ±13	68 ±15*	67 ±15**	63 ±16 *†	<0.001
E/A ratio (1/1)	Norwegians	1.16 ±0.4	0.91 ±03 *	0.98 ±0.4 *	1.11 ±0.5†	<0.001
	Russians	1.23±0.4	0.98 ±0.3*	1.07 ±0.*†	1.10 ±0.4 *†	<0.001
MV E DT (ms)	Norwegians	167 ±40	182 ±54 *	180 ±59	168 ±37 †	<0.001
	Russians	197 ±40	215 ±45*	213 ±39*	106 ±45	<0.001
Ejection fraction (%)	Norwegians	57.6 ±4.8	53.9 ±7.8*	55.8 ±5.7*	48.7 ±9.2*†‡	<0.001
	Russians	57.7 ±4.5	55.6 ±6.0*	57.5 ±4.9†	53.0 ±7.6*†‡	<0.001

Peak velocity: speckle tracking derived left ventricular basal velocity; LA: left atrial; MV: mitral valve; S: systolic; E: early diastolic; A: at atrial contraction;

Bonferoni post-hoc analysis: * p<0.05 for difference towards group A ; †p<0.05 for difference towards group B; ‡ p<0.05 for difference towards group C

Supplemental Table S3: Comparison of functional systolic and diastolic parameters between Russians and Norwegians

	Unadjusted Linear Regression			Adjusted Linear Regression			
	Norwegians	Russians		Norwegians	Russians		
	Unadjusted Mean ± SD	Unadjusted Mean ± SD	p-value	Adjusted mean	Adjusted mean	p-value	Adjusted Mean Difference (CI)
n	828	1044		788	676		
Longitudinal ES strain (%)	**-20.4 ±2.8**	**-19.7 ±2.9**	**<0.001**	-20.2	-20.2	0.892	-0.03 (-0.05 to 0.001)
Longitudinal peak SR S	**-1.20 ±0.20**	**-1.18 ±0.20**	**0.06**	-1.20	-1.17	0.063	-0.03 (-0.05 to 0.001)
Longitudinal peak SR E	**1.57 ±0.33**	**1.53 ±0.35**	**0.005**	1.57	1.55	0.933	0.00 (-0.04 to 0.04)
Longitudinal peak SR A	1.11 ±0.27	1.11 ±0.27	0.893	**1.13**	**1.07**	**<0.001**	**0.06 (0.03 to 0.09)**
Longitudinal displacement (mm)	**16.5 ±2.7**	**15.5 ±2.8**	**<0.001**	16.2	16.0	0.295	0.19 (-0.16 to 0.54)
Peak velocity s´ (cm/s)	**-5.4 ±1.4**	**-5.3 ±1.6**	**0.112**	5.37	5.30	0.613	-0.05 (-0.23 to 0.14)
Peak velocity e´ (cm/s)	**-6.4 ±2.0**	**-6.2 ±2.1**	**0.053**	-6.39	-6.30	0.122	0.18 (-0.05 to 0.40)
Peak velocity a´ (cm/s)	**-6.3 ±1.4**	**-6.2 ±1.4**	**0.032**	**-6.34**	**-6.25**	**0.003**	**-0.25 (-0.42 to -0.09)**
LA volume index (ml/m^2)	24.7 ±7.8	24.8 ±8.8	0.689	**24.7**	**25.7**	**0.003**	**-1.01 (-2.01 to -0.01)**
n	914	1165		872	766		
Heart rate‡	**61.1 ±10.0**	**65.0 ±10.4**	**<0.001**	**61.0**	**64.1**	**<0.001**	**-2.52 (-3.74 to -1.31)**
Stroke volume (ml)	**89.1 ±27.6**	**82.9 ±27.6**	**<0.001**	**89.1**	**83.1**	**0.020**	**3.20 (0.51 to 5.90)**
MV E/e´ ()	**-11.0 ±9.1**	**-11.7 ±10.2**	**0.096**	-10.9	-11.3	0.955	0.04 (-1.21 to 1.28)
MV E velocity (m/s)	**0.65 ±0.17**	**0.67 ±0.17**	**0.148**	0.67	0.68	0.406	-0.01 (-0.03 to 0.01)
MV A velocity (m/s)	**0.65 ±16**	**0.64 ±15**	**0.044**	**0.65**	**0.63**	**<0.001**	**0.04 (0.03 to 0.06)**
E/A ratio ()	**1.07 ±0.39**	**1.10 ±0.36**	**0.059**	**1.07**	**1.13**	**<0.001**	**-0.08 (-0.12 to -0.04)**
MV E deceleration time (ms)	**172 ±45**	**207 ±44**	**<0.001**	**173**	**207**	**<0.001**	**-36.3 (-41.9 to -30.7)**
Ejection fraction (%)	**54.9 ±7.4**	**55.8 ±6.2**	**0.004**	**55.0**	**55.9**	**0.001**	**-2.37 (-3.07 to -1.67)**

Linear Regression Model adjusted for age, sex, BMI, height, systolic BP (blood pressure), diastolic BP, heart-rate, atrial fibrillation, smoking, pulmonary artery pressure, serum values for total, LDL and HDL cholesterol, triglycerides, creatinine, high-sensitive C-reactive protein and HbA1c. ‡ Not corrected for Heart Rate. ES: end-systolic; SR: strain rate; S: systolic; E: early diastolic; A: at atrial contraction; PSS: post-systolic shortening; MV: mitral valve; LA: left atrial; TR: tricuspid regurgitation

Supplemental Table S4: Intra- and inter-investigator variability for strain-based variables by limits of agreement (LOA) and Intra Class Correlation (ICC)

		Russians (n=26)					Norwegians (n=12)					ALL (n=38)
		MEAN	SD	LOWER LOA	UPPER LOA	ICC	MEAN	SD	LOWER LOA	UPPER LOA	ICC	ICC
Intraobserver	Longitudinal ES strain (%)	-0.25	1.33	-2.86	2.36	0.96	-0.4	1.37	-3.09	2.29	0.96	0.96
	Longitudinal peak SR S	-0.01	0.06	-0.13	0.11	0.97	-0.01	0.08	-0.17	0.15	0.93	0.96
	Longitudinal peak SR E	0.05	0.15	-0.24	0.34	0.96	-0.003	0.19	-0.38	0.37	0.72	0.93
	Longitudinal peak SR A	0.000	0.08	-0.16	0.16	0.98	-0.03	0.13	-0.28	0.22	0.98	0.98
	Peak velocity s´ (cm/s)	0.01	0.34	-0.66	0.68	0.99	-0.06	0.43	-0.90	0.78	0.97	0.98
	Peak velocity e´ (cm/s)	-0.02	0.45	-0.90	0.86	0.98	0.01	0.68	-1.32	1.34	0.96	0.97
	Peak velocity a´ (cm/s)	0.09	0.31	-0.52	0.70	0.99	-0.28	0.76	-1.77	1.21	0.94	0.97
	Longitudinal displacement (mm)	0.18	1.02	-1.82	2.18	0.97	0.12	1.22	-2.27	2.51	0.86	0.96
Interobserver	Longitudinal ES strain (%)	1.61	1.6	-1.53	4.75	0.93	1.21	1.59	-1.91	4.33	0.94	0.93
	Longitudinal peak SR S	0.03	0.09	-0.15	0.21	0.94	0.001	0.08	-0.16	0.16	0.95	0.94
	Longitudinal peak SR E	-0.16	0.32	-0.79	0.47	0.80	-0.09	0.3	-0.68	0.50	0.62	0.77
	Longitudinal peak SR A	-0.03	0.16	-0.34	0.28	0.88	0.002	0.17	-0.33	0.34	0.95	0.92
	Peak velocity s´ (cm/s)	0.12	0.63	-1.11	1.35	0.95	0.37	0.62	-0.85	1.59	0.95	0.95
	Peak velocity e´ (cm/s)	-0.13	0.39	-0.89	0.63	0.99	-0.06	0.36	-0.77	0.65	0.99	0.99
	Peak velocity a´ (cm/s)	-0.45	0.59	-1.61	0.71	0.96	-0.05	0.5	-1.03	0.93	0.97	0.96
	Longitudinal displacement (mm)	0.64	1.13	-1.57	2.85	0.96	0.67	1.24	-1.76	3.10	0.87	0.94

ICC: intraclass correlation; myo: myocardial; SR: strain rate; S: strain rate at peak systole; E: strain rate at early diastole; A: strain rate at atrial contraction

Supplemental Table S5: Intra- and interinvestigator variability for conventional echocardiographic parameters comparing the Russian and Norwegian reading group

	Mean	SD	Lower limit	Upper limit	ICC
Ejection fraction (%)	-5.84	7.22	-19.99	8.31	0.77
Stroke volume (ml)	-1.13	17.66	-35.74	33.48	0.81
MV A velocity (cm/s)	-0.004	0.05	-0.10	0.09	0.98
MV E DT (ms)	-17.03	65.61	-146	112	0.79
MV velocity E (cm/s)	0.02	0.07	-0.12	0.16	0.96
E/A ratio (1/1)	0.04	0.11	-0.18	0.26	0.98

MV: mitral valve; A: atrial contraction ; E: early diastole; DT deceleration time; Norwegian – Russian mean

Supplemental Table S6: Covariates in the linear regression model

	Clinical parameters										Bloodtests						
	Age	Sex	BMI	Height	SBP	DBP	HR	AF	TR Vmax	smoking	Total Cholesterol	LDL	HDL	Trigl	Creatinin	HS-CRP	HBA1C
Longitudinal ES strain (%)	0.079	**<0.001**	**<0.001**	0.061	0.247	**0.001**	**0.012**	0.320	0.967	0.293	0.808	0.850	0.519	0.749	0.657	0.525	0.426
Longitudinal peak SR S	**0.002**	0.137	0.134	0.131	0.267	0.533	**<0.001**	0.519	0.564	0.612	0.535	0.583	0.154	0.374	0.833	0.937	0.738
Longitudinal peak SR E	**<0.001**	**<0.001**	**<0.001**	0.619	**0.003**	**<0.001**	0.217	0.214	**0.002**	0.688	0.644	0.617	0.391	0.905	0.384	0.186	0.695
Longitudinal peak SR A	**<0.001**	0.345	0.059	0.419	**0.004**	**<0.001**	**<0.001**	**0.012**	**0.010**	**<0.001**	0.105	0.075	0.379	0.382	0.084	0.468	0.974
Longitudinal displacement (mm)	0.930	**0.022**	**<0.001**	**<0.001**	**0.018**	**0.002**	**<0.001**	**<0.001**	0.161	0.376	0.744	0.826	0.518	0.770	0.484	0.092	**0.029**
Peak velocity s´ (cm/s)	**<0.001**	0.079	**<0.001**	**<0.001**	0.488	0.172	**0.001**	**0.024**	0.910	0.212	0.974	0.831	0.710	0.955	0.381	0.318	0.735
Peak velocity e´ (cm/s)	**<0.001**	**0.004**	**<0.001**	0.367	0.334	**<0.001**	**<0.001**	0.671	**<0.001**	0.480	0.281	0.298	0.396	0.452	0.479	0.719	0.445
Peak velocity a´ (cm/s)	**<0.001**	**<0.001**	0.360	0.055	0.676	**0.009**	**<0.001**	**<0.001**	**0.025**	**0.008**	0.224	0.198	0.259	0.420	0.725	0.213	0.051
LA volume Index (ml/m^2)	0.407	0.056	**<0.001**	**<0.001**	**<0.001**	**<0.001**	**<0.001**	**0.033**	**<0.001**	0.872	0.768	0.557	0.384	0.373	0.178	**0.037**	**0.017**
Heart rate	**0.004**	**0.001**	**<0.001**	**<0.001**	0.124	**<0.001**		**<0.001**	**<0.001**	**<0.001**	**<0.001**	**0.013**	**<0.001**	**<0.001**	**<0.001**	**<0.001**	**<0.001**
Stroke volume (ml)	0.100	**0.001**	**<0.001**	**<0.001**	**0.010**	0.091	**<0.001**	0.660	**0.022**	0.360	0.885	0.781	0.542	0.631	0.638	0.839	0.090
MV E/e´()	**<0.001**	**<0.001**	**<0.001**	0.078	**<0.001**	0.247	**<0.001**	**<0.001**	0.338	**0.001**	0.052	**0.015**	0.165	0.084	0.765	**0.009**	0.075
MV velocity E (cm/s)	**<0.001**	**<0.001**	0.075	**0.003**	**<0.001**	**<0.001**	**<0.001**	**<0.001**	**<0.001**	0.611	0.645	0.603	0.878	0.314	0.317	0.159	**0.008**
MV A velocity (cm/s)	**<0.001**	**0.020**	**<0.001**	**0.001**	**<0.001**	0.138	**<0.001**	0.456	0.061	0.063	**0.017**	**0.009**	**0.003**	**0.002**	0.609	0.594	0.538
E/A ratio ()	**<0.001**	**0.013**	**<0.001**	0.785	0.056	**<0.001**	**<0.001**	**<0.001**	**<0.001**	0.211	**0.030**	**0.021**	**0.018**	0.057	0.702	0.114	0.019
MV deceleration time (ms)	**<0.001**	0.916	0.163	**<0.001**	0.311	**0.015**	0.098	0.102	0.520	**0.008**	0.606	0.502	0.965	0.769	**0.049**	0.122	0.382
Ejection fraction (%)	0.227	**<0.001**	**<0.001**	0.158	**0.005**	**<0.001**	**<0.001**	**<0.001**	0.063	**<0.001**	0.061	**<0.001**	**0.006**	**0.029**	**<0.001**	**<0.001**	**<0.001**

Red and bold are co-variates with significant association with the variable of interest.
ES: endsystolic; SR: strain rate; S: systolic; E: early diastolic; A: at atrial contraction; LA: left atrial; MV: mitral valve
